# Mercy Hospital—Dr. N. S. Davis

**Published:** 1876-02

**Authors:** N. S. Davis


### Clinics.

### MERCY HOSPITAL.

*Clinic by* PROF. N. S. DAVIS, *December* 11, 1875.

### PARTIAL PARALYSIS AND DISEASE OF SPINE.

CASE I. The first case presented to the class was a laboring man, aged about 28 years, naturally strong and muscular. He stated that he had been subject to occasional attacks of asthma for several years, and last week had considerable cough and soreness in his chest, which had been relieved by an expectorant mixture from the Dispensary.

His present complaint is of general weakness, with pain, more or less acute, in the right side, sometimes extending to the shoulder, and which he attributes to "liver complaint." By a systematic questioning, and physical examination by the professor, the history and symptoms of his case were more fully and accurately elicited, as follows: About two years since, the patient first began to feel a numbness and prickling sensation in his toes and feet, which has continued, with steadily increasing weakness more especially in the right foot and leg ; dull pain in the dorsal portion of the spine, with difficulty of raising himself from a stooping position, and some unsteadiness in walking. During the last six months he has had pains, sometimes acute, in the right side, extending from the right margin of the epigastrium along the lower margin of the ribs, and up to the lower angle of the right scapula. There were occasional slighter pains taking the same direction in the left side. He also complained of a distinct sense of numbness and constriction, like a band around the body, just along the lower line of the epigastric and hypochondriac regions. There was well marked tenderness to pressure over the fourth, fifth and sixth dorsal vertebræ, with slight prominence of the spinous processes, but neither auscultation nor percussion elicited any evidence of pulmonary disease, or of alterations in the viscera of the abdomen. There was some tendency to constipation, with impairment of digestion. After calling attention specially to such symptoms as were of direct diagnostic value, the professor remarked that the partial anæsthesia of the right foot, leg and side, with impairment of muscular motion, pointed to disease in some part of the cerebro-spinal nervous centres, while the definite sense of numbness, like a band around the trunk of the body, and the free, healthy condition of the upper extremities, equally point to some part of the spinal cord below the junction of the cervical with the dorsal portion of the spine, as the special seat of trouble. These facts, with the tenderness to pressure and percussion over the middle part of the dorsal portion of the spine, led to the conclusion that the patient was laboring under a chronic grade of inflammation of the meninges and connective tissue of that portion of the spinal cord, more especially the right half. And from the length of time since the first symptoms were noticed, it was inferred that some degree of thickening and hypertrophy had taken place in those structures, with atrophy of the nerve cells.

The prospect of recovery depends upon the extent of these changes, and the ability of the patient to comply with the conditions most important in the management of his case.

*Treatment.* Rest in a recumbent position as much of the time as possible; moderate, but persistent, counter-irritation over the affected portion of the spine; alteratives with anodynes internally for the first two weeks, and subsequently the more tonic class of iodides, constituted the treatment recommended.

CASE II. The attention of the class was next called to a little girl, aged five years, who had first been presented to the class four weeks previously, with well marked paralysis of the portio-dura of the seventh pair of nerves on the right side of the face. At the time of her first presentation in the clinic, her mother stated that about four weeks previously, the child had been attacked with acute pain in the right mastoid region, accompanied by local tenderness and some swelling, with considerable general fever. The pain and fever disappeared in a few days, but slight swelling over the mastoid process and under the ear of the right side, with hyperæsthesia of the scalp over nearly all of that side of the head, and complete loss of expression in the same side of the face, remained. The child received no medical treatment, and continued in the condition just mentioned, until her first visit to the clinic four weeks since. At that time, the right upper eyelid drooped a little, and the lids failed to close during sleep; an effort at laughing caused the mouth to be drawn largely to the left; and there was a little swelling, apparently from periosteal thickening, over the right mastoid process, and hyperæsthesia in touching the hair or scalp over the right temporal and right half of the occipital regions. In all other respects the child appeared well. The diagnosis then made was, that the child had been attacked with subacute inflammation of the periosteum covering the mastoid part of the temporal bone and adjacent areolar tissue, accompanied by sufficient plastic exudation to cause thickening of the inflamed structures, and pressure upon the trunk of the portio-dura nerve, where it passes out from the cranium. After the first few days, the fever and more acute symptoms subsided, but leaving that nerve paralyzed, and the occipital and temporal branches of the fifth pair involved enough to cause the hyperæsthesia of the right side of the scalp. With this view of the nature of the case, it was stated that the leading indications for treatment were, to remove the slight inflammatory action still remaining, and to cause a more complete disintegration and reabsorption of the exudative material that still pressed injuriously upon the trunk of the nerves. To accomplish these purposes, the following alterative and anodyne mixture was ordered: ℞. Sodi. Iod., ʒ ijss; Hydrarg. Bichlorid., gr. i; Tinct. Conii, ʒ iv; Syrup. Prunus Virg., ʒ iv; Aquæ, ℥ iij. Mix.

The mother was directed to give the child twenty drops in a little water, four times a day, for the first week, and three times a day the second week, and then bring her again to the clinic. This was done, and when she returned at the end of the second week, the only noticeable change was less hyperæsthesia of the right side of the head, more perfect closure of the eyelids during sleep, and no perceptible swelling either behind or beneath the lobe of the ear. Still there was no visible action of the muscles of the right side of the face in laughing. The same prescription was continued three times a day for one week longer, and then reduced to twice a day. During the third week the mother began to notice some expression in the right side of the face, which has steadily increased, until now, at the end of four weeks from the commencement of the treatment, the paralysis is entirely removed, and the child appears well. The class was cautioned against the too prevalent custom of adopting a routine treatment by electricity, strychnine, etc., for every case of paralysis, without due attention to the special pathological conditions causing the paralysis.

